# Perceptions of Clinical Adverse Event Reporting by Nurses and Midwives

**DOI:** 10.3390/healthcare12040460

**Published:** 2024-02-11

**Authors:** Anna Majda, Michalina Majkut, Aldona Wróbel, Anna Kurowska, Agata Wojcieszek, Kinga Kołodziej, Iwona Bodys-Cupak, Joanna Rudek, Krystian Barzykowski

**Affiliations:** 1 Laboratory of Theory and Fundamentals of Nursing, Institute of Nursing and Midwifery, Faculty of Health Sciences, Jagiellonian University Medical College, 12 Michałowskiego Street, 31-126 Krakow, Poland; anna.majda@uj.edu.pl (A.M.); aldona1.wrobel@uj.edu.pl (A.W.); anna2.kurowska@uj.edu.pl (A.K.); agata.wojcieszek@uj.edu.pl (A.W.); kinga1.kolodziej@uj.edu.pl (K.K.); i.bodys-cupak@uj.edu.pl (I.B.-C.); joanna1.rudek@uj.edu.pl (J.R.); 2 Institute of Psychology, Jagiellonian University, 6 Ingardena Street, 30-060 Krakow, Poland

**Keywords:** adverse events, nurses, midwives, perceptions

## Abstract

The level of safety in healthcare units is mainly characterized by the occurrence of medical adverse events. The aim of the study was to present the experiences of reporting clinical adverse events and the perceptions of nurses working in internal medicine wards, surgical wards and midwives on these issues. The cross-sectional survey was conducted from October 2022 to April 2023. The study used the Author’s Survey Questionnaire and sampling by assessment was applied. The study included nurses working in internal medicine wards and surgical wards as well as midwives at nine hospitals in a large provincial city in Poland, amounting to 745 participants. A one-way analysis of variance ANOVA and a post-hoc test (Fisher’s NIR) were used. The significance level (*p*) did not exceed 0.05. Nurses working in surgical wards, internal medicine wards and midwives thought that clinical adverse events should be reported, and perceived this as an important and useful activity in ensuring patient safety. The most common adverse events reported by respondents were falls F(2.742) = 52.07; *p* = 0.001, bedsores F(2.742) = 19.62; *p* = 0.001, patient disappearances F(2.742) = 3.98; *p* = 0.019, and hospital-acquired infections F(2.742) = 3.88; *p* = 0.021. The most frequently selected factors influencing the abandonment of adverse event reporting were excessively complex paperwork, no or little harm to the patient or a fear of the negative consequences. The study suggests that an important way to overcome the barriers to nurses and midwives reporting adverse events would be to create a supportive atmosphere in which they could report errors and the reasons for them honestly and without fear, and to improve the way adverse events are reported at the personal and institutional levels.

## 1. Introduction

There is no universal definition of an adverse event (AE), as the meaning of the term depends on the harm (illness, injury, suffering, disability or death), its character (physical, social or psychological) and the general approach to the problem, especially the legal solutions in this regard, in the different health care systems [1]. Adverse events should not be confused with the natural progression of a disease or predictable therapeutic complications, which are not classified as adverse events because they usually cannot be prevented. According to the definition in the Polish accreditation standards developed by the Centre for Quality Monitoring in Health Care, an adverse event is harm caused during/as a result of treatment, not related to the natural course of the disease, the patient’s health status or the risk of occurrence [2]. Adverse events included in the Polish accreditation standards are situations such as foreign bodies left in the surgical site; an inappropriate patient, operated site, or surgical procedure; catheter-related vessel infection; body damage and sepsis resulting from a surgical procedure; a pulmonary embolism or deep vein thrombosis after a surgical procedure; suicide in hospital; inappropriate administration of a drug (the wrong drug, patient, time of administration, dose or route of administration); a fall in hospital; the untimely delivery of care; reoperations; unplanned repeat hospital admissions; the patient’s arbitrary departure from hospital; and others [2]. Among adverse events, sentinel events deserve special attention. These are events that should never have happened and which have particular consequences (death, serious physical or mental harm). They also include the surrender of a newborn to another family, surgery on the wrong person or the wrong body part, an instrument left behind after surgery or another procedure, a post-transfusion hemolytic reaction caused by transfusion of incompatible blood, radiotherapy of the wrong area of the body or a dose higher than planned [3].

In Poland, the Act on Quality in Healthcare and Patient Safety of 16 June 2023 (Dz.U. RP, Warsaw, 24 August 2023, item 1692) is in force. According to its provisions, directors of entities providing medical services with public money are obliged to establish internal management systems—among other things, to prevent adverse events. On the one hand, the management of adverse events is enforced by the Act; on the other hand, the frequency of their occurrence and the costs incurred because of them can be observed. According to Nowak-Musiej A., in Poland adverse events affect four in 10 patients in outpatient care, 80% of which could be prevented, and 6% of inpatients/hospitalized patients. The costs incurred to remedy the effects of adverse events are 0.2–16.5% of public hospital expenditure [4]. The Council of the European Union (EU) issued recommendations on patient safety in 2009, indicating that Member States should establish, maintain and improve comprehensive adverse event reporting systems and learn from them to monitor the extent and causes of adverse events, and develop effective solutions and patterns of response on this basis. It also recommended the dissemination of patient-directed information on risks, as well as safety measures put in place to prevent errors or harm and to reduce their incidence [1].

The literature highlights that there are many causes of adverse events. Among the most important are the complexity of the treatment processes; the occurrence of latent errors in the health care delivery system; the disproportion between advances in medical knowledge and technology and the ability of medical staff to undergo continuous training; the general weaknesses of human nature; the state of health and efficiency of medical staff; and cognitive errors resulting from the specific functioning of the human brain and sensory organs [5]. Adverse events are caused by various factors, including environmental, team, individual or task factors that affect safety or the lack thereof. Other causes are, for example, the actions of multidisciplinary teams (doctors, nurses or paramedics) under severe time pressure. Adverse events pose a risk to both patients and the entire organization (e.g., the hospital). If the risk materializes, it carries specific consequences. For example, a foreign body left in the surgical field for the patient will result in reoperation, pain and physical suffering or even death; while for the hospital the consequences will be the costs of additional examinations and/or additional surgical intervention, prolonged hospitalization, compensation and loss of reputation [6,7]. The Patient Ombudsman has categorized adverse events reported by patients into clinical, organizational and other events. Clinical adverse events may include injury to the patient during surgery, leaving a foreign body in the patient, errors and inadequacies in diagnosis and treatment, hospital-acquired infections, inadequacies during delivery or failure to provide the patient with the appropriate and/or timely care. Organizational adverse events may relate to patient burns or organizational errors that result in failure to provide appropriate care to the patient. Other adverse events include those related to patient suicide attempts, assaults, violence or patient falls [8].

One method of building a safety system in the realization of medical processes is risk assessment, analysis and management, which in healthcare entities should lead to the prevention of adverse events. Risk management includes both active and passive risk analysis, e.g., by assessing adverse events, but also all aspects of the organization [1]. Due to the number and complexity of diagnostic and therapeutic activities performed in the healthcare system, clinical risk management is important. It is specifically concerned with improving the quality and safety of healthcare services, by identifying situations and circumstances that are risks to the patient, and actions to prevent or control these risks. The risks incurred by a healthcare provider can be divided into some typical areas, by the type of risks and the type of actions taken to minimize them, such as financial risk—the uncertainty of contracting with a public payer; legal risk—referring to changes in legal regulations, claims by patients and their families for adverse events; and operational risk—including all specific risks that directly affect the ability to conduct business in strategic areas, i.e., the ability to maintain business continuity [1].

The level of safety in healthcare units is mainly characterized by the occurrence of medical adverse events. It is the intention of the Authors of this paper to increase interest in addressing patient safety among medical professionals, especially nurses and midwives, with a particular focus on issues that, despite their importance, are not sufficiently considered in the literature. The increasing complexity of healthcare means a greater risk of harm, and requires greater vigilance, focus and increased investment on the part of nurses and midwives. The interest in patient safety on the part of these professional groups must facilitate their understanding of the causes and types of adverse events, help them develop the skills to report and manage errors during healthcare delivery, and provide knowledge of how to prevent and reduce errors and what to do when they make errors, witness an error or when they learn that someone has made an error.

Patient safety in medical treatments is essential to prevent unnecessary risks and harm, ensuring the quality of care. From an ethical and moral perspective, healthcare professionals have a responsibility to provide safe care, respect patient autonomy and act in the patient’s best interest. This ethical commitment to patient safety reflects integrity, respect for life and the need for transparency in communication, including honesty about potential errors. Continuous improvement of practices and learning from mistakes are ethical actions contributing to the establishment of safe and patient-centered healthcare.

The aim of the presented cross-sectional study was to learn about the experiences of clinical adverse events by nurses working in internal medicine and surgical wards and midwives, as well as their involvement in the recognition and reporting of such events, including their perceptions.

Specific objectives:

1. Learning about respondents’ opinions on/perceptions of the reporting of clinical adverse events and their involvement in recognizing and reporting them;
2. Assessment of perception of the importance of reporting clinical adverse events by participants;
3. Assessment of the types of clinical adverse events experienced by respondents;
4. Learning about barriers to adverse event reporting, including the reasons for not reporting adverse events at the personal and institutional level;
5. Assessment of reactions of the participants’ colleagues encountered after reporting the adverse event.

## 2. Materials and Methods

### 2.1. Design and Data Collection

The cross-sectional study is described in detail in an article by Majda et al. [9], as the article presented here is a continuation of the description of the results obtained under the auspices of the same statutory project NR43/DBS/000233. The inclusion criterion was nurses in internal medicine wards and surgical wards, as well as midwives in maternity wards, while the exclusion criterion was nurses and midwives working in primary care or hospital administration. Questionnaires were available in hard copy. They were distributed at nine hospitals located in a large provincial city in Poland, whose management had given permission for the survey to be conducted in the abovementioned wards by the project manager. Then, after one week, the questionnaires were collected from the nurses and midwives who completed them and left them with the ward nurses/midwives in sealed envelopes. The data collected was entered into an Excel file by the research assistants. A total of 3100 survey sheets were distributed. Of the 1100 sheets returned, 745 were completed correctly and included for further analysis. The return rate was 35.48%. The sample was representative of the wider spectrum of nurses and midwives. Judgmental sampling was used in the study. This type of purposive sampling involves individuals selected for inclusion in the study based on the professional judgement of the researcher. It is the opposite of probability sampling, in which individuals are drawn with a specific probability from the population of interest.

### 2.2. Measurement Tools

The study used a diagnostic survey method and a questionnaire technique. The Author’s Survey Questionnaire was used as the research tool. It contained 15 questions (single-choice or multiple-choice) that related to the occurrence and reporting of adverse events, reasons for not reporting, experiences of reporting and the monitoring body for adverse events. The questionnaire included:

- 5 single-choice questions: Should adverse events be reported? Is the reporting of adverse events important and useful? Should the occurrence of adverse events be associated with penalties for medical staff? Does the occurrence of adverse events indicate inadequate care? containing answer options according to a Likert scale (definitely—1 point, rather yes—2 points, rather not—3 points, definitely not—4 points); and the question Should there be an Adverse Event Reporting Unit in the hospital? with the following answer options: yes, no, I don’t know, I don’t have an opinion
- 5 multiple-choice questions: Reporting of adverse events: What type of adverse events have you encountered at work? What are the factors influencing the abandonment of adverse event reporting? Who should be part of the Unit? On what assumptions/principles should the work of the Unit be based?
- 5 questions aimed exclusively at participants who have ever reported an adverse event in the past. Single-choice questions on a Likert scale (definitely—1 point, rather yes—2 points, rather not—3 points, definitely not—4 points): Did you find it difficult to complete the Adverse Event Card? When reporting an adverse event, did you have any concerns about the appropriateness of your actions and your future career? Has your experience with adverse event reporting caused you to refrain from reporting adverse events in the future? Has the reporting of adverse events resulted in negative consequences or penalties? (response options for this question: never—1 point, rarely—2 points, sometimes—3 points, often—4 points, always—5 points). Multiple choice question: Which reactions of your colleagues did you encounter after reporting an adverse event?

The survey questionnaire was prepared in accordance with the guidelines for the use of survey tools to ensure the good quality of the measurement carried out, to avoid erroneous conclusions from results obtained, to allow inter-center studies to be conducted according to a common protocol and to increase the size of the study group in the future. At the stage of preliminary/pilot research, the problem under study was defined, survey items/questions were prepared, the response format was determined and standardization was carried out (the conditions for conducting the survey were defined—the survey was preceded by information on the purpose of the study and concise instructions on how to complete the questionnaire). However, it is important to note that in the present study, we did not utilize a questionnaire in the conventional sense, specifically a psychological questionnaire designed to measure latent traits. Instead, we employed a survey to inquire about individuals’ experiences and opinions related to adverse reporting. Consequently, we did not undertake a standard psychometric validation process, such as computing Cronbach’s alpha, which is commonly employed in the validation of personality questionnaires.

The results obtained on the following sample group, using other research tools such as the Justice Sensitivity Inventory, the Feelings in Moral Situations Scale, the Perceived Stress at Work Scale and the Questionnaire of Attitudes of Healthcare Workers towards Reporting of Clinical Adverse Events (P-RoCAES) were discussed in a separate research item. These tools achieved satisfactory psychometric properties in the validation process for Polish conditions [9,10].

### 2.3. Ethical Considerations

Respondents were presented with the purpose of the study and informed that participation in the study was anonymous, which meant that the answers given would not be attributed to the respondents personally. The confidentiality of the participants was ensured by coding the participants, using as identification numbers the consecutive numbers with which the returned questionnaires were numbered. All electronic files containing personally identifiable information, such as place of work, were password-protected to prevent access by unauthorized users. Participants were informed that participation was entirely voluntary and that completing the questionnaires meant implicit consent. Respondents could refuse to participate or withdraw from the project at any time without any negative consequences. The study, conducted within the framework of the statutory project NR43/DBS/000233, received approval from the Bioethics Committee No. 1072.6120.214.2022. This is the second article within this project [9].

### 2.4. Data Analysis

The data obtained were statistically analyzed using Statistica (version 13). A one-way ANOVA with grouping by ward type and a post-hoc test (Fisher’s NIR) were used. The significance level (*p*) did not exceed 0.05.

## 3. Results

### 3.1. Individuals Surveyed

A total of 745 participants took part in the survey. The characteristics of the study group were presented in Table 1. 

### 3.2. Reporting of Clinical Adverse Events by Internal Medicine Nurses, Surgical Nurses and Midwives—Opinions, Perceptions

Table 2 presents the means and standard deviations for the rate of agreement with the statement in the multiple-choice questions (the rate ranges from 0, indicating no agreement, to 1, indicating perfect agreement) or the degree to which the respondents agreed with the statement (questions on scales 1–4 or 1–5). Nurses working in surgical wards, internal medicine wards and midwives thought that adverse events should be reported, and viewed this as an important and useful activity. Respondents were against punishing medical staff for adverse events. More than 80% of the respondents stated that reporting clinical adverse events ensures patient safety; more than 70% that it ensures the safety of the medical staff; and 50–60% thought that it may result in preventive measures (the differences between the respondents in this area were significant, with the lowest level of agreement with the given statement among midwives). About 10% of the respondents considered that reporting adverse events is associated with negative consequences at work, threatening their career (the differences between respondents in this area were significant, with the lowest level of agreement with the given statement among midwives).

### 3.3. Types of Clinical Adverse Events Experienced by Internal Medicine Nurses, Surgical Nurses and Midwives

Table 3 shows that the most common adverse events encountered by the respondents were falls, bedsores, patient disappearances and hospital-acquired infections, while the least common were transfusion errors, failure to provide care, deaths, suicide attempts and suicides. Hospital-acquired infections and bedsores were significantly least frequently encountered by midwives and most frequently by surgical nurses. Midwives were significantly more likely to declare that they did not encounter adverse events at all, and significantly less likely to encounter patient falls, patient injury, death or missing patients. In response to the question “Does the occurrence of adverse events indicate inadequate care?”, the respondents mostly answered that this was rather not the case, and did not differ in this aspect. The most frequently selected factors influencing the abandonment of adverse event reporting, in the opinion of the respondents, were too much/complicated paperwork (albeit significantly less frequent among internal medicine nurses), fear of negative consequences and no or little harm to the patient. A significantly more frequent factor influencing the abandonment of adverse event reporting in the opinion of surgical nurses was the lack of witnesses to the event. The vast majority of respondents (more than 70%) declared that there should be an Adverse Event Reporting Unit in the hospital. When asked who should be part of the Unit to which adverse events could be reported, most respondents indicated a trusted person/leader/coordinator nominated from among the staff to report and analyze events and a ward nurse (significantly, midwives were most often of this opinion). Most respondents felt that the Unit should base its work on assumptions/principles such as (1) allowing voluntary, unfettered reporting of adverse events to all staff on paper and/or electronically rather than identifying reporters; (2) improving care rather than initiating disciplinary action against reporters; and (3) analyzing the reported adverse event (how and why it happened) and generating remedial solutions rather than seeking answers as to who was at fault. There were no significant differences among internal medicine nurses, surgical nurses and midwives in this issue.

Table 4 shows which reactions the respondents encountered from their colleagues after reporting the adverse event. The most common reaction reported by the respondents after reporting an adverse event was to show support for such behavior. There were significant differences in an indifferent attitude/no reaction (most often among midwives) and experiencing support for such an action (most often among internal medicine nurses, and least often among midwives). For those respondents who declared that they report adverse events, filling in the Adverse Event Card was mostly not perceived to be difficult, although midwives scored significantly lowest. Similarly, in response to the question of whether, when reporting an adverse event, respondents had concerns about the rightness of their actions and their future career, overall respondents stated that they rather had no such concerns, albeit midwives scored significantly lowest. This was comparable to the question “Has the reporting of adverse events resulted in negative consequences, penalties?“—respondents mostly answered never or rarely, but midwives scored significantly highest. To the question “Has your experience with adverse event reporting caused you to refrain from reporting adverse events in the future?”, the majority of respondents answered that it rather has not, with midwives scoring the lowest.

## 4. Discussion

The nurses and midwives who took part in the survey felt that adverse events should be reported. They described it as an important and useful activity that should not involve penalties for medical staff. Studies by Alanazi et al. (a systematic review of nine studies) and Kakemam et al. (a cross-sectional survey of 2295 nurses) also point to the need to promote a non-punitive response to adverse event reporting [11,12]. Such a systems approach may result in medical staff being more willing to report adverse events [13]. It is worth mentioning that the willingness to report adverse events also depends on good communication, the working environment, staff experience and teamwork [11,12,14,15]. Teamwork can result in a reduction in the incidence of adverse events and an increase in the percentage of adverse events reported [16]. Respondents in the presented study were usually supported by their colleagues. Additionally, they reported that their experience of reporting adverse events was unlikely to cause them to give up reporting.

The nurses who participated in the study by Hodak et al. said that errors can lead to positive improvements. On the other hand, the nurses emphasized that errors reflect on them and that they sometimes feel that they are the ones being reported on and not the event [17]. In contrast, a study by Araujo et al. showed that, according to nurses, reporting adverse events provides information on how to prevent events, contributes to the process of investigating the causes of events and improves the quality of care [18]. The vast majority of our respondents said that reporting adverse events ensures the safety of patients as well as medical staff. According to a study by Kakemam et al., a high perception of patient safety is associated with a lower incidence of adverse events [12]. Respondents were most likely to experience adverse events such as falls, hospital-acquired infections and bedsores. Similar results occurred in a study by Kang et al. [19]. Zanetti et al. pointed to pharmacological events and hospital-acquired infections [20]. In contrast, in a study by Labrague L.J., the most common adverse events reported were complaints from patients and their families [21]. It is worth mentioning that a positive attitude towards safety in healthcare may result in a reduction in adverse events such as falls, medication errors, hospital-acquired infections or deaths [11].

Respondents to the survey felt that the occurrence of adverse events was unlikely to be indicative of inadequate care. However, according to a study by Nantsupawat et al., an adverse event in the form of a lack of or inadequate care may be linked to excessive patient-to-staff ratios and lack of resources, and may also increase the risk of further adverse events [22]. In an analysis by Kawalec-Kajstura et al., more than half of the respondents (59.1%) were of the opinion that staffing was insufficient to ensure patient and nurse safety [23]. In their study, Aiken et al. demonstrated that adherence to nurse staffing standards reduced the incidence of patient deaths [24]. According to Leńczuk-Gruby et al., the main causes of adverse events are an insufficient number of medical staff and work overload. In this study, a small number of nurses working in surgical wards admitted to being the perpetrator of adverse events, which, according to the authors, indicated that staff are still afraid to report such incidents in the workplace. Prevention of adverse events, in the opinion of the respondents in this study, should consist of providing more staffing per patient and focusing on the job [25].

Factors influencing the reluctance to report were identified, such as documentation complexity, the perception of minimal harm to the patient or a fear of negative consequences. The last reason seems surprising, as overall respondents reported that they were unlikely to have concerns about the rightness of their behavior, for which they were unlikely to face punishment or other negative consequences. Similar results occurred in a study by Mansouri et al., where respondents cited reporting barriers such as a fear of the consequences, procedural and management barriers [26]. On this basis, it can be concluded that there is a need to define precisely how to report. It may be helpful to establish an adverse event reporting unit to enable voluntary, unfettered reporting of adverse events and analysis of incidents, thereby contributing to improved care without penalizing reporters. According to Miller et al., it is also worth considering the use of tools to improve reporting [27].

The vast majority of respondents (more than 70%) in the presented survey also declared that there should be an adverse event reporting unit in the hospital. When asked who should be part of the unit to which adverse events could be reported, most respondents indicated a trusted person/leader/coordinator appointed from among staff to report and analyze events (e.g., doctor, nurse) and a ward nurse (significantly most often midwives); the fewest respondents suggested the director and his/her deputy. According to Leńczuk-Gruby et al., in order for positive changes to occur in terms of reducing the incidence of adverse events, nursing staff should report all incidents they have perpetrated or witnessed. On the other hand, it is up to the management to create an environment and atmosphere in medical facilities where those who report negligence and medical errors will feel understood and personally safe [25].

The first study conducted by the Society for the Promotion of Healthcare Quality showed that it was not possible to determine a specific number of adverse events occurring in the healthcare system. However, this was not due to a lack of such incidents, but to an inadequate system for monitoring adverse events [28]. In a study by Aftyka et al., as many as 84% of nurses admitted that they had committed a medical error at least once in their professional practice [29]. An even higher result was obtained in a 2015 study conducted by the Polish Society for Quality of Healthcare, in which approximately 90% of doctors and nurses admitted to having committed a medical error [30]. Different results were obtained by Kawalec-Kajstura et al., with 32.5% of 83 respondents declaring that an adverse event had occurred, while 47% had witnessed one [23]. In the present study, only 23% of the nurses and midwives surveyed did not encounter an adverse event.

In summary, the survey conducted presents perceptions of adverse events and their reporting, and describes barriers and experiences of reporting in a group of nurses and midwives. This information, collected from a relatively large number of individuals, can provide a reference for further efforts to improve patient safety in healthcare. The study provides valuable information on developing practices and policies to promote effective adverse event reporting in the hospital setting, ultimately helping to improve patient safety. Nevertheless, the study is characterized by some limitations, namely sampling by assessment, unequal study groups, a limited study area and possible errors in respondents’ answers, which shows the need for further research in the field of medical staff perceptions of adverse events.

## 5. Conclusions

The study suggests that: (1) nurses working in surgical wards, internal medicine wards and midwives believe it is necessary to report clinical adverse events and see this as an important and useful activity; (2) the most common adverse events encountered by respondents were falls, hospital-acquired infections and bedsores; and (3) creating a supportive environment for nurses and midwives to openly report adverse events and improving reporting processes at the personal and institutional levels are key strategies for overcoming barriers to reporting.

## Figures and Tables

**Table 1:** Characteristics of the study group.

Characteristic		%	N
Gender	Women	95.84	714
	Men	4.16	31
Age (years)	21–30	26.44	197
	31–40	18.26	136
	41–50	25.37	189
	51–60	26.71	199
	61–67	3.22	24
Workplace	Internal medicine wards	61.34	457
	Surgical wards	22.55	168
	Maternity wards	16.11	120
Education	Bachelor’s degree	33.96	253
	Master’s degree	49.53	369
	Diploma (in nursing or midwifery)	15.97	119
	No data available	0.54	4

**Table 2:** Means and standard deviations in terms of answers given to individual questions on adverse events for the three groups of respondents (surgical nurses, internal medicine nurses and midwives).

Questionnaire Questions	Surgical Nurses	Internal Medicine Nurses	Midwives	ANOVA Test
Should adverse events be reported? (scale of 1–4, where 1 means definitely and 4 means definitely not)	1.16 (0.41)	1.17 (0.47)	1.12 (0.32)	F(2.742) = 0.72; *p* = 0.489
Is the reporting of adverse events important and useful? (scale of 1–4, where 1 means definitely and 4 means definitely not)	1.23 (0.47)	1.23 (0.50)	1.18 (0.39)	F(2.742) = 0.40; *p* = 0.670
Reporting of adverse events… (multiple-choice question)				
Ensures patient safety	0.87 (0.34)	0.81 (0.39)	0.82 (0.38)	F(2.742) = 1.41; *p* = 0.246
Ensures the safety of medical staff	0.77 (0.42)	0.77 (0.42)	0.79 (0.41)	F(2.741) = 0.13; *p* = 0.878
Involves additional documentation	0.63 (0.48)	0.58 (0.49)	0.60 (0.49)	F(2.742) = 0.80; *p* = 0.450
Is the duty of medical staff	0.74 (0.44)	0.71 (0.46)	0.68 (0.47)	F(2.741) = 0.53; *p* = 0.589
Threatens careers	0.09 (0.29) ^A^	0.06 (0.24) ^B^	0.02 (0.13) ^A,B^	F(2.742) = 3.23; *p* = 0.040
Involves negative consequences at work	0.10 (0.29)	0.09 (0.28)	0.06 (0.24)	F(2.742) = 0.66; *p* = 0.518
May result in changes to prevent adverse events	0.65 (0.48) ^A,B^	0.61 (0.49) ^A^	0.50 (0.50) ^B^	F(2.741) = 3.53; *p* = 0.030
Should the occurrence of adverse events be associated with penalties for medical staff? (scale of 1–4, where 1 means definitely and 4 means definitely not)	3.48 (0.77)	3.57 (0.67)	3.63 (0.59)	F(2.742) = 1.67; *p* = 0.189

Comments: Standard deviations (SD) are given in brackets. Means with the same letter (e.g., A,A) within a row are significantly different from each other (*p* < 0.05). In terms of multiple-choice statements, the table presents the statement support index—it ranges from 0, indicating no support, to 1, indicating perfect support for the option. F—Fisher’s NIR test result; *p*—level of statistical significance.

**Table 3:** Types of clinical adverse events, factors influencing the abandonment of their reporting and rules of the Adverse Event Reporting Unit as perceived by respondents.

Questionnaire Questions	Surgical Nurses	Internal Medicine Nurses	Midwives	ANOVA Test
What type of adverse events have you encountered at work? (multiple-choice question)				
Incorrect identification of the patient	0.43 (0.50)	0.34 (0.47)	0.36 (0.48)	F(2.742) = 2.32; *p* = 0.099
Medication errors	0.45 (0.50)	0.40 (0.49)	0.44 (0.50)	F(2.742) = 0.78; *p* = 0.460
Transfusion errors	0.14 (0.34)	0.12 (0.32)	0.10 (0.30)	F(2.742) = 0.46; *p* = 0.631
Hospital-acquired infections	0.63 (0.49) ^A,B^	0.51 (0.50) ^A^	0.48 (0.50) ^B^	F(2.742) = 3.88; *p* = 0.021
Bedsores	0.57 (0.50) ^A^	0.52 (0.50) ^B^	0.23 (0.42) ^A,B^	F(2.742) = 19.62; *p* = 0.001
Falls	0.76 (0.43) ^A^	0.77 (0.42) ^B^	0.32 (0.47) ^A,B^	F(2.742) = 52.07; *p* = 0.001
Body damage	0.23 (0.42)	0.27 (0.44) ^A^	0.16 (0.37) ^A^	F(2.742) = 3.34; *p* = 0.036
Patient disappearances	0.24 (0.43) ^A^	0.24 (0.43) ^B^	0.13 (0.33) ^A,B^	F(2.742) = 3.98; *p* = 0.019
Failure to provide care	0.05 (0.21)	0.08 (0.27)	0.06 (0.24)	F(2.742) =0.91; *p* = 0.403
Deaths	0.15 (0.36)	0.12 (0.32)	0.07 (0.25)	F(2.742) = 2.32; *p* = 0.099
Suicide attempts	0.10 (0.29)	0.09 (0.29)	0.08 (0.28)	F(2.742) = 0.06; *p* = 0.940
Suicides	0.04 (0.19)	0.04 (0.19)	0.03 (0.16)	F(2.742) = 0.28; *p* = 0.755
I have not encountered any such event	0.01 (0.11) ^A^	0.03 (0.18) ^B^	0.17 (0.37) ^A,B^	F(2.742) = 22.52; *p* = 0.001
Does the occurrence of adverse events indicate inadequate care? (scale of 1–4, where 1 means definitely and 4 means definitely not)	3.03 (0.83)	2.92 (0.82)	3.00 (0.71)	F(2.742) = 1.25; *p* = 0.287
What are the factors influencing the abandonment of adverse event reporting? (multiple-choice question)				
Too much/complicated paperwork	0.62 (0.49) ^A^	0.52 (0.50) ^A,B^	0.62 (0.49) ^B^	F(2.742) = 3.98; *p* = 0.019
Fear of negative consequences	0.58 (0.49)	0.58 (0.49)	0.67 (0.47)	F(2.742) = 1.67; *p* = 0.189
Indifference to adverse events	0.21 (0.41)	0.17 (0.38)	0.14 (0.35)	F(2.742) = 1.34; *p* = 0.262
Supervisors do not require the reporting of adverse events	0.05 (0.23)	0.08 (0.27)	0.07 (0.26)	F(2.742) = 0.51; *p* = 0.602
No or little harm to the patient	0.40 (0.49)	0.44 (0.50)	0.39 (0.49)	F(2.742) = 0.78; *p* = 0.460
No witnesses to the incident	0.22 (0.42) ^A,B^	0.13 (0.34) ^A^	0.11 (0.31) ^B^	F(2.742) = 4.62; *p* = 0.010
Lack of time	0.31 (0.46)	0.35 (0.48)	0.37 (0.49)	F(2.742) = 0.73; *p* = 0.481
No sense of responsibility for an adverse event	0.24 (0.43)	0.21 (0.41)	0.20 (0.40)	F(2.742) = 0.41; *p* = 0.664
Lack of a unit responsible for reporting adverse events	0.07 (0.25)	0.06 (0.23)	0.07 (0.25)	F(2.742) = 0.13; *p* = 0.879
Should there be an Adverse Event Reporting Unit in the hospital?				
Yes	72.46%	75.44%	73.33%	N/A
No	1.80%	3.51%	4.17%	
I don’t know	17.96% ^A,B^	11.40% ^A^	8.33% ^B^	
I don’t have an opinion	7.78%	9.65%	14.17%	
Who should be part of the Unit? (multiple-choice question)				
Director/Deputy Director	0.21 (0.41)	0.18 (0.38)	0.18 (0.38)	F(2.742) = 0.43; *p* = 0.649
Head Nurse	0.30 (0.46)	0.35 (0.48)	0.37 (0.49)	F(2.742) = 1.16; *p* = 0.313
Head of Department/Dispensary/Facility	0.37 (0.48)	0.42 (0.49)	0.37 (0.49)	F(2.742) = 1.03; *p* = 0.356
Ward Nurse	0.42 (0.50) ^A^	0.49 (0.58)	0.58 (0.50) ^A^	F(2.742) = 3.27; *p* = 0.039
A trusted person/leader/coordinator nominated from among the staff to report and analyze events (e.g., doctor, nurse)	0.74 (0.44)	0.66 (0.47)	0.63 (0.49)	F(2.742) = 2.40; *p* = 0.091
On what assumptions/principles should the work of the Unit be based? (multiple-choice question)				
Allowing voluntary, unfettered reporting of adverse events to all staff on paper and/or electronically rather than identifying reporters	0.72 (0.45)	0.68 (0.47)	0.68 (0.47)	F(2.742) = 0.47; *p* = 0.628
Improving care rather than initiating disciplinary action against reporters	0.65 (0.48)	0.73 (0.45)	0.64 (0.48)	F(2.742) = 2.68; *p* = 0.070
No identification of the personnel involved in the adverse event.	0.15 (0.36)	0.16 (0.36)	0.15 (0.36)	F(2.742) = 0.05; *p* = 0.955
Working independently of other systems, e.g., complaints system, criminal and professional liability system	0.20 (0.40)	0.19 (0.39)	0.13 (0.34)	F(2.742) = 1.18; *p* = 0.309
Analyzing the reported adverse event (how and why it happened) and generating remedial solutions rather than seeking answers as to who was at fault	0.68 (0.47)	0.70 (0.46)	0.63 (0.48)	F(2.742) = 1.13; *p* = 0.323
Anonymity/confidentiality of the participants in the incident	0.32 (0.47)	0.30 (0.46)	0.38 (0.49)	F(2.742) = 1.63; *p* = 0.198
No consequences for the participants in the incident	0.23 (0.42)	0.25 (0.43)	0.24 (0.43)	F(2.742) = 0.06; *p* = 0.946
Determining the form of adverse event reporting (development of a procedure)	0.43 (0.50)	0.46 (0.50)	0.38 (0.49)	F(2.742) = 1.21; *p* = 0.298

Comments: Standard deviations (SD) are given in brackets. Means with the same letter (e.g., A,A) within a row are significantly different from each other (*p* < 0.05). In terms of multiple-choice statements, the table presents the statement support index—it ranges from 0, indicating no support, to 1, indicating perfect support for the option. F—Fisher’s NIR test result; *p*—level of statistical significance; N/A—not applicable.

**Table 4:** Reactions of colleagues to the reporting of clinical events as perceived by the nurses and midwives surveyed. Questions addressed only to persons who have ever reported an adverse event in the past.

Questionnaire Questions	Surgical Nurses	Internal Medicine Nurses	Midwives	ANOVA Test
Did you find it difficult to complete the Adverse Event Card? (scale of 1–4, where 1 means definitely and 4 means definitely not)	2.79 (0.74) ^A^	2.74 (0.79) ^B^	2.51 (0.75) ^A,B^	F(2.591) = 3.89; *p* = 0.021
Which reactions of your colleagues did you encounter after reporting an adverse event? (multiple-choice question)				
Indifferent attitude/no reaction	0.27 (0.45) ^A^	0.29 (0.46) ^B^	0.40 (0.49) ^A,B^	F(2.723) = 3.02; *p* = 0.049
Undermining the rightness of an adverse event report	0.11 (0.32)	0.12 (0.33)	0.14 (0.35)	F(2.723) = 0.27; *p* = 0.764
Showing support for such behavior	0.44 (0.50) ^A^	0.54 (0.50) ^A,B^	0.33 (0.47) ^B^	F(2.723) = 9.65; *p* = 0.001
When reporting an adverse event, did you have any concerns about the appropriateness of your actions and your future career? (scale of 1–4, where 1 means definitely and 4 means definitely not)	2.97 (0.84) ^A^	2.99 (0.79) ^B^	2.61 (0.87) ^A,B^	F(2.591) = 8.06; *p* = 0.001
Has the reporting of adverse events resulted in negative consequences or penalties? (scale of 1–5, where 1 means never and 5 means always)	1.60 (0.86) ^A^	1.52 (0.82) ^B^	1.89 (0.98) ^A,B^	F(2.592) = 6.82; *p* = 0.001
Has your experience with adverse event reporting caused you to refrain from reporting adverse events in the future? (scale of 1–4, where 1 means definitely and 4 means definitely not)	3.22 (0.66) ^A^	3.12 (0.72) ^B^	2.92 (0.79) ^A,B^	F(2.592) = 4.54; *p* = 0.011

Comments: Standard deviations (SD) are given in brackets. Means with the same letter (e.g., A,A) within a row are significantly different from each other (*p* < 0.05). In terms of multiple-choice statements, the table presents the statement support index—it ranges from 0, indicating no support, to 1, indicating perfect support for the option. F—Fisher’s NIR test result; *p*—level of statistical significance.

## Data Availability

Data are contained within the article.
